# Supplementary material for: Chasing genetic structure in coralligenous reef invertebrates: patterns, criticalities and conservation issues
Source: Sci Rep. 2018 Apr 11;8:5844. doi: 10.1038/s41598-018-24247-9 (PMC5895814; doi:10.1038/s41598-018-24247-9)

The following supplement accompanies the article

Chasing genetic structure in coralligenous reef invertebrates: patterns, criticalities and conservation issues

RUNNING HEAD: Connectivity and conservation of coralligenous reefs

Federica Costantini[1, 2,3*,] Filippo Ferrario[4], Marco Abbiati[1, 2, 3, 5]

1 Dipartimento di Scienze Biologiche, Geologiche e Ambientali, Università di Bologna, UOS Ravenna

2 Centro Interdipartimentale di Ricerca per le Scienze Ambientali, Università di Bologna, Via S. Alberto 163, I – 48123 Ravenna, Italy

3 CoNISMa, Piazzale Flaminio 9, 00197 Roma, Italy

4 Québec-Océan, Université Laval, Québec, QC, Canada

5 Consiglio Nazionale delle Ricerche, Istituto di Scienze Marine, ISMAR, Via P. Gobetti 101, 40129 Bologna, Italy

Supplementary methods

Combinations of keywords in the "Topic" field used to systematically search the literature from 2000 to 2016: "mediterranean" AND ["population genetic*" OR "population genetic structur*" OR "genetic structur*" OR "phylogeography" OR "dispersal"] AND ["molecular marker*" OR "microsatellite*" OR "mitochondrial" OR "allozyme*"] AND "marine invertebrate*". Additionally, to ensure the inclusion of relevant articles on typical species of coralligenous habitat, the focus of our research, the main query was refined by replacing "marine invertebrate*" in turn with 1) ["gorgonian*" OR "coral" OR "cnidaria*" OR "anthozoa*"], 2) ["echinoderm*" OR "sea star*"], 3) ["sponge* OR "porifer*"], and 4) ["tunicata*" OR "ascidia*"]. Six additional records identified through other sources were added (Figure 6).

Data extraction:

Each entry in the full dataset included the following variables (Supplementary Table S1): (i) taxa analysed, (ii) dispersal potential (low vs. high), (iii) mating system (brooders vs. broadcaster), (iv) nutritional mode of the larvae (lecitotrophic, planktotrohic, direct development), (v) maximum pelagic larval duration (when data were not available in the analysed paper we obtained this information from other publications on the same species; see Supplementary data Table S2), (vi) molecular markers used, (vii) sampling area considered (ranked in four groups: Atlantic-Mediterranean transition, Mediterranean scale (thousand of kilometres), regional scale (hundreds of kilometres), local (tens of kilometres) and micro scale (meters)), (viii) total number of the populations, (ix) maximum geographic distance at Mediterranean scale, (x) global F_{ST} as carried out from each study, (xi) slope and standard errors of the regression line between pairwise F_{ST} and geographical distance. Pairwise F_{ST} were transformed using the formula by Baco et al.[1] and the slope of the regression were calculated using transformed F_{ST}, (xii) pattern of spatial genetic structure, and (xiii) basin from where the pairwise F_{ST} values were extrapolated.

Quantitative assessment of the genetic structure within the Mediterranean Sea: Meta-analyses

The observation from Ledoux et al.[2] appeared to be an outlier within META-set because its ES standard error was at least an order of magnitude smaller than the others, conferring it with an excessive weight, and it was therefore excluded from the analysis (IBD slope ± standard error = 0.0079 ± 0.0008).

We first we analysed the effect of the single factors "Marker" and "Maximum PLD" and their additive effect. We did not include the interaction term because PLD values were unevenly distributed between levels of the factor "Marker". We then tested the effect of "Phylum", excluding the only observation belonging to tunicates. Because of a potential confounding effect in the analysis due to the overlap between groups "Marker" and "Phylum", the last analysis was performed only after verifying the absence of a marker effect.

Since the Cochran's Q-test for heterogeneity for the null model was significant, we always used random effects models (where residual heterogeneity is calculated and tested against a χ^2-distribution with n-1 degrees of freedom[3]; $Q_{df\,30}$= 147.69, $P < 0.0001$ for META-set; $Q_{df\,20}$= 127.17, $P < 0.0001$ for META-set for long PLD only). All models were fitted using the most popular DerSimonian-Laird estimator[3] and AIC was used to choose among competing models.

ANCOVA analysis

We did not include the interaction "Maker × Maximum Distance" because geographical distances within mtDNA groups were almost all clustered between 300 and 600 km (distance range 0 – 1200 km).

Contrary to meta-analyses, we did not test the factor "Phylum" in the ANCOVA, as the potential confounding effect of "Marker" could not be excluded because of its significance in terms that included it (either alone or in interaction, see Results).

Models were first fitted with maximum likelihood methods to allow model selection, based on AICc and significance assessment, via LRT. The best model was then refitted using the restricted maximum likelihood.

R Packages used

The package *metafor* [4] was used for meta-analyses while *nlme*[5] for ANCOVA. Model selection was performed using packages *AICcmodavg* [6] and *glmulti*[7].

References

1. Baco, A. R. *et al.* A synthesis of genetic connectivity in deep-sea fauna and implications for marine reserve design. *Mol. Ecol.* **25,** 3276–3298 (2016).
2. Ledoux, J.-B. *et al.* Genetic survey of shallow populations of the Mediterranean red coral [*Corallium rubrum* (Linnaeus, 1758)]: new insights into evolutionary processes shaping nuclear diversity and implications for conservation. *Mol. Ecol.* **19,** 675–90 (2010).
3. Borenstein, M., Hedges, L. V., Higgins, J. P. T. & Rothstein, H. R. *Introduction to Meta-Analysis*. (2009).
4. Viechtbauer, W. Conducting Meta-Analyses in R with the metafor Package. *J. Stat. Softw.* **36,** 1–48 (2010).
5. Pinheiro, J., Bates, D., DebRoy, S., Sarkar, D. & Team, R. C. nlme: Linear and nonlinear mixed effects models. *R Packag. version* **3,** 96 (2017).
6. Mazerolle, M. J. AICcmodavg: Model selection and multimodel inference based on (Q)AIC(c). (2017).
7. Calcagno, V. glmulti: Model selection and multimodel inference made easy. R package version 1.0.7. (2013).

Supplementary Table S2. Dataset of marine invertebrate pelagic larval duration and their references.

Species	Pelagic larval duration (PLD)	Reference
Corallium rubrum	4-12 d	Vighi (1972)
Eunicella singularis	122 d	Theodor (1967); Weinberg and Weinberg 1979
Eunicella cavolinii	4-12 d	Costantini F, Personal communication
Paramuricea clavata	6-23 d	Linares et al. (2008)
Astroides calycularis	2-3 d	Goffredo et al. (2010)
Cladocora caespitosa	2-3 d	Goffredo et al. (2010)
Leptopsamnia pruvoti	7-42 d	Goffredo et al. (2005)
Balanophyllia europaea	7-42 d	Goffredo and Zaccanti (2004)
Echinaster sepositus	3 d	Bailly and Flammang (2008)
Marthasterias glacialis	127 d	Barker and Nichols (1983)
Amphipholis squamata	absent	Boissin et al. (2008)
Ophioderma longicauda	weeks	Boissin et al. (2011)
Ophiothrix fragilis	21–26 d	Perez-Portela et al. (2013)
Chondrosia reniformis	2-3 d	Uriz et al. (1998)
Crambe crambe	2-3 d	Maldonado and Bergquist (2002)
Paraleucilla magna	2-3 d	Uriz et al. (1998)
Phorbas fictitius	2-3 d	Uriz et al. (1998)
Scopalina lophyropoda	2-3 d	Uriz et al. (1998)
Ircinia fasciculata	2-3 d	Uriz et al. (1998)
Spongia lamella	2-3 d	Maldonado and Bergquist (2002)
Spongia officinalis	2-3 d	Maldonado and Bergquist (2002)
Botryllus schlosseri	hours	Svane and Young (1989)
Pseudodistoma crucigaster	10 h	Tarjuelo et al. (2004)
Clavelina lepadiformis	2-3 d	Svane and Young (1989)
Cystodytes dellechiajei	10 h	Tarjuelo and Turon (2004)
Halocynthia papillosa	2 weeks	Kim et al. 2012
Pycnoclavella communis	hours	Svane and Young (1989)

References

Bailly, P., & Flammang, P. Fine structure of the lecithotrophic *Brachiolaria* larvae in two spinulosid sea stars. 8th Larval Biology Symposium, Lisbon (2008).

Barker, M. F., & Nichols, D. Reproduction, recruitment and juvenile ecology of the starfish, *Asterias rubens* and *Marthasterias glacialis*. *J. Mar. Biol. Ass. U.K.* **63**, 745–765 (1983).

Boissin E., Feral J. P., & Chenuil A. Defining reproductively isolated units in a cryptic and syntopic species complex using mitochondrial and nuclear markers: the brooding brittle star, *Amphipholis squamata* (Ophiuroidea). *Mol. Ecol.* **17**, 1732-1744 (2008).

Boissin, E., Stöhr, S., & Chenuil, A. Did vicariance and adaptation drive cryptic speciation and evolution of brooding in *Ophioderma longicauda* (Echinodermata: Ophiuroidea), a common Atlanto-Mediterranean ophiuroid?. *Mol. Ecol.* **20**, 4737-4755 (2011).

Goffredo, S., Gasparini, G., Marconi, G., Puntignano, M. T., Pazzini, C., & Zaccanti, F. Gonochorism and planula brooding in the Mediterranean endemic orange coral *Astroides calycularis* (Scleractinia: Dendrophylliidae): morphological aspects of gametogenesis and ontogenesis. *Mar. Biol. Res.* **10**, 421–436 (2010).

Goffredo, S., Radetić, J., Airi, V., & Zaccanti F. Sexual reproduction of the solitary sunset cup coral *Leptopsammia pruvoti* (Scleractinia, Dendrophylliidae) in the Mediterranean. 1. Morphological aspects of gametogenesis and ontogenesis. *Mar. Biol.* **147**, 485-495 (2005).

Goffredo, S., & Zaccanti, F. Laboratory observations on larval behaviour and metamorphosis in the Mediterranean solitary coral *Balanophyllia europaea* (Scleractinia, Dendrophylliidae). *Bul. Mar. Sci.* **74**, 449-457 (2004).

Kim, W. J., Lee, C. I., Kim, H. S., Han, H. S., Jee Y. J., et al. Population genetic structure and phylogeography of the ascidian, *Halocynthia roretzi*, along the coasts of Korea and Japan, inferred from mitochondrial DNA sequence analysis. *Biochem. Syst. Ecol.* **44**, 128–135 (2012).

Linares, C., Coma, R., Mariani, S. et al. 2008b. Early life history of the Mediterranean gorgonian *Paramuricea clavata*: implications for population dynamics. *Invertebr. Biol.* **127**, 1–11 (2008b).

Maldonado, M., & Bergquist P. R. Phylum Porifera. Atlas of marine invertebrate larvae (ed. by CM Young), 21–50. (2002).

Pérez-Portela, R., Almada, V. & Turon, X. Cryptic speciation and genetic structure of widely distributed brittle stars (Ophiuroidea) in Europe. *Zool. Scri.* **42**, 151–169 (2013).

Svane, I., & Young, C. M. 1989. The ecology and behaviour of ascidian larvae. *Oceanogr. Mar. Biol.* **27**, 45-90 (1989).

Tarjuelo, I., & Turon, X. Resource allocation in ascidians: reproductive investment vs. other life-history traits. *Invertebr. Biol.* **123**,168-180 (2004).

Tarjuelo, I., Posada, D., Crandall K. A., et al. Phylogeography and speciation of colour morphs in the colonial ascidian *Pseudodistoma crucigaster*. *Mol. Ecol.* **13**, 3125-3136 (2004).

Theodor, J. Contribution a l'étude des gorgones VII. Ecologie et comportement de la planula. *Vie Milieu* 18, 291–301 (1967).

Uriz, M. J., Maldonado, M., Turon, X., & Martí, R. How do reproductive output, larval behaviour, and recruitment contribute to adult spatial patterns in Mediterranean encrusting sponges? *Mar. Ecol. Progr. Ser.* **167**, 137–148 (1998).

Vighi, M. Etude sur la reproduction du *Corallium rubrum* (L). *Vie Milieu* **23,** 21–32 (1972).

Weinberg, S., & Weinberg, F. The life cycle of a gorgonian: *Eunicella singularis* (Esper, 1794). *Bijdr Dierkd* **48,** 127–140 (1979).

Supplementary Table S3. Estimate dispersal distance for each species that demonstrated significant IBD using the method of Kinlan and Gaines (2003). For *Corallium rubrum* and *Crambe crambe* we used the already published values of mean dispersal distance calculated by Calderon et al. (2007)* and Ledoux et al. (2010)** using an "individual based" sampling. To calculate the estimated dispersal scale we used the formula used by Chust et al. (2016): dispersal distance = 0.0016 (IBD slope)$^{-1.0001}$).

Species	IBD	Slope IBD	Estimated dispersal distance (m)	References
Crambe crambe	yes	-	0.2	from Calderon et al. (2007)*
Corallium rubrum	yes	-	0.3	from Ledoux et al. (2010)**
Astroides calycularis	yes	0.0208	76.95	Casado-Amezua et al. (2012)
Ophioderma longicauda C5	yes	0.0275	58.21	Weber et al. 2015
Paramuricea clavata	yes	0.0382	41.9	Pilczynska et al. (2016)
Eunicella cavolinii	yes	0.0184	86.99	Masmoudi et al. (2016)
Ophiothrix fragilis	yes	0.0059	271.32	Taboada & Perez-Portela (2016)

References:

Calderon, I., Ortega, N., Duran, S., Becerro, M., Pascual, M., & Turon, X. Finding the relevant scale: clonality and genetic structure in a marine invertebrate (*Crambe crambe*, Porifera). *Mol. Ecol.* **16**, 1799-1810 (2007).

Casado-Amezúa, P., Goffredo, S., Templado, J., & Machordom, A. Genetic assessment of population structure and connectivity in the threatened Mediterranean coral *Astroides calycularis* (Scleractinia, Dendrophylliidae) at different spatial scales. *Mol. Ecol.* **21**, 3671-3685 (2012).

Chust , et al., Dispersal similarly shapes both population genetics and community patterns in the marine realm. *Sci. Rep.* **6** (2016).

Ledoux, J. B., Garrabou, J., Bianchimani, O., Drap, P., FÉRAL, J. P., & Aurelle, D. (2010). Fine-scale genetic structure and inferences on population biology in the threatened Mediterranean red coral, *Corallium rubrum*. *Mol. Ecol.* **19**, 4204-4216 (2010).

Masmoudi, M. B., Chaoui, L., Topçu, N. E., Hammami, P., Kara, M. H., & Aurelle, D. Contrasted levels of genetic diversity in a benthic Mediterranean octocoral: Consequences of different demographic histories?. *Ecol. Evol.* **6**, 8665-8678 (2016).

Pilczynska, J., Cocito, S., Boavida, J., Serrão, E., & Queiroga, H. Genetic diversity and local connectivity in the Mediterranean red gorgonian coral after mass mortality events. *PloS one* **11**, e0150590 (2016).

Kinlan, B., Gaines, S. Propagule dispersal in marine and terrestrial environments: a community perspective. Ecology 84, 2007–2020 (2003).

Taboada, S., & Pérez-Portela, R. Contrasted phylogeographic patterns on mitochondrial DNA of shallow and deep brittle stars across the Atlantic-Mediterranean area. *Sci. Rep*. **6** (2016).

Weber, A. A. T., Mérigot, B., Valière, S. & Chenuil, A. Influence of the larval phase on connectivity: Strong differences in the genetic structure of brooders and broadcasters in the *Ophioderma longicauda* species complex. *Mol. Ecol.* **24,** 6080–6094 (2015).

Supplementary Figure S1. Boxplots of maximum geographical distance per Phylum. Each boxplot describes the variability of the recorded maximum distance for each study included in the ANCOVA and/or Meta analysis. The thick line in the box represents the median, the box includes the 1st and 3rd quantile and the dotted lines extend to the minimum and maximum values. For each Phylum it is reported the sample size (N) and the distance range in km. Tunicates are presented for the sake of completeness, as they were not included in the meta-analysis testing the phylum effect.

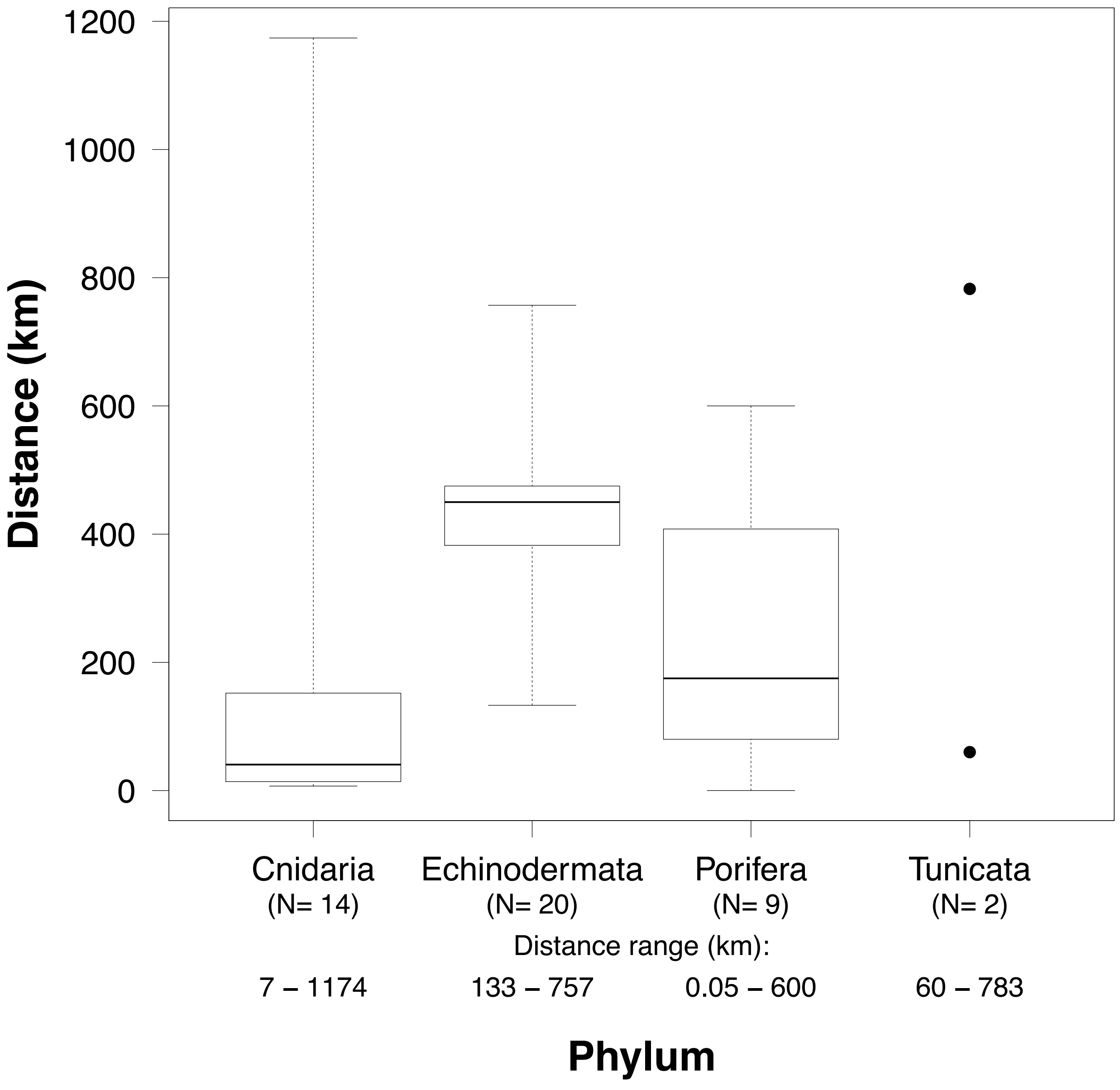

Supplement: Supplementary file 1 — Supplementary data [file 41598_2018_24247_MOESM1_ESM.pdf]
